# Relative Importance of Deterministic and Stochastic Processes in Driving Arbuscular Mycorrhizal Fungal Assemblage during the Spreading of a Toxic Plant

**DOI:** 10.1371/journal.pone.0095672

**Published:** 2014-04-18

**Authors:** Guoxi Shi, Yongjun Liu, Lin Mao, Shengjing Jiang, Qi Zhang, Gang Cheng, Lizhe An, Guozhen Du, Huyuan Feng

**Affiliations:** MOE Key Laboratory of Cell Activities and Stress Adaptations, School of Life Sciences, Lanzhou University, Lanzhou, China; Institute for Plant Protection (IPP), CNR, Italy

## Abstract

Both deterministic and stochastic processes are expected to drive the assemblages of arbuscular mycorrhizal (AM) fungi, but little is known about the relative importance of these processes during the spreading of toxic plants. Here, the species composition and phylogenetic structure of AM fungal communities colonizing the roots of a toxic plant, *Ligularia virgaurea*, and its neighborhood plants, were analyzed in patches with different individual densities of *L. virgaurea* (represents the spreading degree). Community compositions of AM fungi in both root systems were changed significantly by the *L. virgaurea* spreading, and also these communities fitted the neutral model very well. AM fungal communities in patches with absence and presence of *L. virgaurea* were phylogenetically random and clustered, respectively, suggesting that the principal ecological process determining AM fungal assemblage shifted from stochastic process to environmental filtering when this toxic plant was present. Our results indicate that deterministic and stochastic processes together determine the assemblage of AM fungi, but the dominant process would be changed by the spreading of toxic plants, and suggest that the spreading of toxic plants in alpine meadow ecosystems might be involving the mycorrhizal symbionts.

## Introduction

Understanding ecological processes that drive community assembly is one of the key ecological topics. Niche-based theory emphasizes the importance of deterministic processes (e.g. environmental filtering and competitive exclusion) in structuring community [1], whereas neutral theory suggests that community assembly is mainly determined by the stochastic processes [2]. Although the two theories are obviously different, both of them have been successfully employed in explaining the diversity and assemblage of community. In recent years, more and more ecologists have observed that the native communities are controlled by both deterministic and stochastic processes, suggesting that one community composition at any point should be the result of interplay of dispersal limitation, environmental filtering and species interaction [3], [4]. Nonetheless, the current knowledge of community assembly is mainly derived from plant communities [5], [6], whether those unseen communities, such as soil microorganisms, also follow these assembled rules needs further investigations.

Soil microorganisms such as bacteria, fungi and nematodes, are very important in regulating biogeochemical cycles [7]. Among these microorganisms, arbuscular mycorrhizal (AM) fungi, which can form obligately mutualistic associations with the roots of most terrestrial plants, are especially renowned because they can efficiently promote plant nutrient uptake [8], improve the stress tolerance of their host plants [9] and influence many ecosystem processes [10]. Recent evidence has shown that the ecological function of an AM fungal community is highly dependent on the species composition [11], [12], so that exploring the assembly mechanisms of AM fungal community is essential before their functioning in the fields can be well predicted and even controlled. Previous studies have shown that the deterministic factors, such as soil nutrient availability [13], [14], light intensity [15], pH [16], phenology [17], [18] and host plant identity [19]–[21], play key roles in structuring AM fungal community. However, the stochastic processes, such as dispersal limitation [22] and priority effects [23], [24], can also exert great impacts on the community assembly of AM fungi. So far, the assembly mechanisms of AM fungal community have been well elucidated using species abundance model [23], randomization test [25], neutral model [26] and bipartite network analysis [27]. In these studies, however, AM fungal species are often considered as phylogenetically equivalent entities, and the influence of phylogenetic relatedness between species on fungal community assembly is also neglected. In recent decades, there has been a growing consensus to merge phylogenetic relatedness between species with the studies of community ecology, namely the phylogenetic analysis of community structure (hereafter referred to as phylogenetic analysis) [28], [29]. This method can provide great promise for the study of community assembly, and demonstrate the importance of phylogenetic relatedness between species in driving community assembly. It is a key step to clarify whether the functional traits of studied organism are conserved (the functional traits are similar among closely related species) or not when conducting a phylogenetic analysis. Under the assumption of phylogenetic conservatism, in theory, the environmental filtering is expected to generate pattern of phylogenetical clustering (co-occurring species are more related than expected by chance); conversely, competitive exclusion should generate phylogenetic overdispersed (co-occurring species are more distantly related than expected by chance) [4], [30]. The stochastic processes should generate a phylogenetically random community [4], [30]. As the functional traits of AM fungi (e.g. ability of hyphal colonization in both roots and soils) are conserved [31], [32], the phylogenetic analysis might be effective, and by which the assembly mechanisms of AM fungal community have been partly elucidated by recent studies [31], [33], [34].

*Ligularia virgaurea* (Asteraceae), a perennial toxic plant for livestock, is an indicator species of degraded meadows on the Qinghai-Tibetan Plateau [35]. In the past decades, large spreading of *L. virgaurea* in this region has resulted in serious loss of ecological and economic services. The large spreading of *L. virgaurea* is partly attributed to its aggressive sexual and asexual reproduction [35] and exudation of allelochemicals [36], but whether the spreading of *L. virgaurea* is involving their associated soil microbes remains unclear. To fully understand whether the AM fungi have a potential role in promoting the *L. virgaurea* spreading, it is required to examine the AM dynamics and explore the ecological processes driving the AM fungal assemblage across a spreading gradient, because changes of AM fungal assemblage would result in great shifts of plant-plant interaction and plant communities [37], [38].

Recent studies have suggested that some toxic plants would change the interactions between their surrounding plants and belowground AM fungi using phytochemicals [39], [40], and these changes could facilitate the growth of themselves [39], [41]. Therefore, we hypothesized that the spreading of *L. virgaurea*, like other toxic plants, would change community assemblage of AM fungi in its neighborhood plant roots. To test this hypothesis, we chose four patches with different individual densities of *L. virgaurea* in an alpine meadow ecosystem, and the AM fungal communities in the roots of *L. virgaurea* and its neighborhood plants were analyzed using DNA cloning and sequencing techniques. In particular, the objectives of this study were to address the following questions: 1) how do the assemblage of AM fungi in the roots of *L. virgaurea* and its neighborhood plants respond to the spreading of *L. virgaurea*? 2) Which ecological process (environmental filtering, competitive exclusion or stochastic process) is more important in driving the community assembly of AM fungi during the spreading of *L. virgaurea*?

## Materials and Methods

### Ethics statement

The field site is supported by Lanzhou University, China. No specific permits are required for this study, and also our field works did not involve endangered or protected species.

### Filed site and sampling procedure

This study was conducted in an alpine meadow at the Maqu experimental site of the Research Station of Alpine Meadow and Wetland Ecosystems of Lanzhou University, which is located in the eastern Qinghai-Tibet Plateau of China (33°44′N, 101°52′E; 3570 m a.s.l.). The climate of this region is cold humid-alpine with a mean annual temperature of 1.2°C (11.5°C maximum in July and −10.0°C minimum in January) and a mean annual precipitation of 650 mm. The vegetation in this region consists mainly of arctic, alpine and Chinese-Himalayan flora, and is dominated with *Kobresia capillifolia* (Cyperaceae), *Elymus nutans* (Gramineae) and *Anemone rivularis* (Ranunculaceae). The growing season is from May to September.

Four patches with different individual densities of *L. virgaurea*, including no *L. virgaurea* (Control), low density (LD), moderate density (MD) and high density (HD), were selected within a 50×50 m flat meadow on 24 August, 2012. These four patches could well represent different spreading degrees of *L. virgaurea*. Small and flat research field selected in this study could ensure that the original vegetation and soil conditions were relatively homogeneous. In each patch, we randomly selected five 0.5×0.5 m quadrats. The individual numbers and heights of *L. virgaurea* and the plant species composition in each quadrat were measured. In order to determine the AM fungal community colonizing *L. virgaurea* roots in each patch, three individuals of *L. virgaurea* were excavated randomly in each quadrat, and their fine roots were collected and pooled as one sample. In each quadrat, five soil cores (diameter: 3.8 cm, depth: 25 cm) were taken randomly, mixed adequately as one sample in a plastic bag and transported to laboratory in an ice box. All roots excluding *L. virgaurea* were separated carefully from each soil sample and mixed thoroughly as one sample to determine AM fungal community composition in the roots of neighborhood plants. The *L. virgaurea* roots were distinguished from other plant roots according to their morphological character, color and special smell. In order to describe easily, all plants in Control patch were also called neighborhood plants even there was no *L. virgaurea* in this patch. Each root sample was divided into three subsamples, one for determining AM fungal colonization, one for measuring the root N and P concentration, and the remaining one for DNA extraction. The percent root length colonization by AM fungi (%RLC), arbuscular colonization (%AC) and vesicular colonization (%VC) were quantified using the magnified intersection method [42]. All individual plants in each quadrat were clipped to the soil surface and grouped into five groups: sedge, grasses, legume, forb and *L. virgaurea*. All shoots were dried at 80°C for 48 h, and weighed. All dry shoots of *L. virgaurea* and non-*L. virgaurea* plants in each quadrat were grounded to fine powders for determining tissue nutrient concentration, respectively.

### Analysis of soil characteristics and plant N and P concentration

Soil moisture content was measured gravimetrically after drying at 105°C for 24 h. Soil pH was measured in 1 M KCl (1∶5 w/v) using a pH electrode. Soil organic carbon and total N were analyzed by the CHNS-analysis system (Elementar Analysen Systeme, GmbH, Hanau, Germany) with the burning method at 450°C and 1250°C, respectively. The concentration of soil available N (NO_3_-N+NH_4_-N) was measured using a FIAstar 5000 Analyzer (FOSS, Hillerød, Denmark) after extraction with 2 M KCl (1∶5 w/v). Soil available P was extracted following the Mehlich-3 method and measured using the molybdate-blue colorimetrical method [43]. Plant N content was analyzed by the San^++^ Continuous Flow Analyzer (Skalar Analytical B.V., Breda, Netherlands) using the semi-micro Kjeldahl method. Plant P content was measured using the molybdate-blue colorimetric method after digestion with sulfuric acid [44].

### Molecular analysis

DNA was extracted from 35 root samples (20 neighborhood plant and 15 *L. virgaurea* root samples, 100 mg fine roots for each sample) using a Plant DNA Extraction Kit (Tiangen Biotech, Beijing, China) following the manufacture's instruction. Partial SSU rDNA fragments of AM fungi were amplified via nested PCR with a first primer pair GeoA2-Geo11 [45] and a second primer pair NS31-AML2 [46], [47] using the Taq PCR Kit (New England Biolabs, MA, USA). The PCR conditions and mixtures of both the first and second amplification were the same as the method described by Liu et al. [14]. The second PCR products were purified using the AxyPrep DNA Gel Extraction Kit (Axygen Inc, Hangzhou, China), and the expected DNA products (*c.* 560 bp) were obtained. Thirty-five purified DNA samples were ligated into pGEM-T vector (Promega, WI, USA) and transformed into *Escherichia coli* DH5α according to manufacture's instructions, resulting in 35 clone libraries. For each clone library, 48 putative positive transformants were selected randomly and used to prepare the plasmid templates using the freezing and thawing method [19]. Inserted DNA fragments were re-amplified with primer pair NS31-AML2 using the same condition as the second PCR. A total of 1572 positive PCR products was obtained from 35 clone libraries and then screened using restriction fragment length polymorphism (RFLP) with the restriction enzymes *Hin*fI and *Hin*1II (Fermentas, Vilnius, Lithuania). Digested products were examined on 2.5% (w/v) agarose gels with ethidium bromide staining, and the RFLP patterns were only compared within one sample. One representative clone of each RFLP type in each sample was sequenced using vector primer T7 by the Major Biotech Company (Shanghai, China), for a total of 435 sequences. The remaining clones were classified by RFLP patterns and the clone numbers of each RFLP/sequence type were recorded. All DNA sequences were edited and compared with public nucleotide sequence databases using the online BLAST search tool (http://blast.ncbi.nlm.nih.gov/Blast.cgi), and those with low bit scores and high *E*-values were suspected as chimeras [48]. Non-AM fungal sequences and chimeric sequences were eliminated from the dataset before further analysis. All AM fungal sequences were multiple aligned using Clustal W [49], and delimited to species-level groups (each species-level group was regarded as an AM fungal phylotype) according to 97% sequence similarity using the furthest neighbor algorithm in the Mothur program [50]. The most closely related sequences from GenBank database and the representative sequences of major families of Glomeromycota were aligned using the program Clustal W [49], and the neighbor-joining tree was constructed using the MEGA 5.0 with the Kimura two-parameter model [51]. The classification of our AM fungal phylotypes was according to the phylogeny and taxonomy of Glomeromycota (http://schuessler.userweb.mwn.de/amphylo/). All representative sequences were submitted to the GenBank database under the accession number KF467255-KF467324.

### Statistical analysis

All statistical analyses were performed using SPSS 13.0 (SPSS Inc., Chicago, IL, USA) or R version 2.15.2 (http://www.r-project.org/). All data were tested for normality after appropriate transformation and tested for equality of variance using Levene's test. The community composition of AM fungi colonizing roots was calculated on the basis of the clone numbers of each phylotype in a root sample. To ensure the abundance of dominant and rare phylotypes contributed equally to the resultant matrix, the data of all AM fungal communities were Hellinger-transformed using the function of “decostand” from “vegan” library of R package [52]. Effects of patch or individual density of *L. virgaurea* on measured plant and AM fungal variables were tested by one-way ANOVA, and their significant differences were determined using Tukey's honestly significant difference test at the 95% confidence level (*P*≤0.05). Sampling effort curves of the richness of AM fungal phylotypes (Mao Tau) were computed using EstimateS 8.0 [53].

To elucidate the relationships between AM fungal communities and environmental variables, all measured plant and soil variables (see Table S1–S3 in File S1) were fitted as vectors onto the non-metric multidimensional scaling (NMDS) plots of AM fungal communities, which was measured with the Bray-Curtis dissimilarity, using the function of ‘envfit’ from ‘vegan’ library of the R package [52]. We also tested the correlations between AM fungal communities and the matrices of plant species composition, soil characteristics, N and P variables using Mantel test, in which the matrices of AM fungal communities and environmental datasets were represented by the Bray-Curtis and Euclidean distance, respectively [52]. To determine the indicator species of AM fungi for each patch, we conducted indicator species analyses (species with Indval values ≥0.6 are strong indicators) for each AM fungal community using the function ‘indval’ from the ‘labdsv’ library of the R package [54].

To test whether the species abundance distribution (SAD) of our AM fungal community (community data for *L. virgaurea* and neighboohood plants, respectively) complies with expectation by chance, we fitted Etienne's neutral model using the maximum likelihood estimates of metacommunity diversity (θ) and the immigration parameter (I) [55]. Based on the abundance distribution of our detected phylotypes, we used the PARI/GP algorithm to calculate the θ and I, and then run a sequential construction scheme that generates 200 expected species abundance distributions that are consistent with the neutral theory [55]. The SAD within the real and simulated data sets was grouped using the function “preston” from the R package [56], and the goodness-of-fit of our data to the neutral model was assessed using *χ^2^* test and Kolmogorov-Smirnov Z test (in these tests, high *P* values indicate that the data fit the model well).

To examine the relative importance of spatial effect and environmental factors in regulating AM fungal community composition, we partitioned the Beta-diversity into spatial and environmental components according to the methods described by Legendre et al. [57], [58]. The spatial structures of the samples were modeled using principal coordinates of neighborhood matrix (PCNM), which is based on the calculation of a principal coordinate analysis (PCoA) of a truncated distance matrix [59]. PCNM eigenfunctions represent a spectral decomposition of the spatial relationships among samples, and can be considered as purely spatial variables in an ordination-based analysis [16]. Forward section of PCNM variables was performed, based on 999 permutation tests (α = 0.05), using the “packfor” library of R package [60]. The variation of the community composition data was then partitioned between the extracted PCNM spatial variables and the soil environmental variables (Table S3 in File S1) using the function “varpart” from the “vegan” library of R package [52]. The total variations of community compositions across four patches were divided into four fractions: (a) variation explained by the environmental variables and not spatial structured, (b) variation explained by the environmental variables and spatial structured, (c) spatially structured variation not explained by the environmental variables, and (d) residual variation [58].

The phylogenetic analysis was performed to explore the phylogenetic patterns of our AM fungal communities using the “picante” library of R package [61]. The mean nearest taxon distance (MNTD) was chosen to quantify the phylogenetic relatedness of co-occurring species. We calculated the MNTD based on 999 randomizations of the “taxa.labels” null model (shuffle distance matrix labels), and the mean nearest taxon distances for each species were weighted by species abundance. To determine whether the phylogenetic structure of our observed community is different from the null community, the nearest taxon index (NTI) was calculated based on the differences between the observed metric and mean metric of the null communities. The NTI represents a standardized effect size of MNTD in communities, and was calculated using the following formula: NTI = −1×[MNTD_observed_−MNTD_random_]/SD(MNTD_random_), where SD represents the standard deviation [31]. The significant differences between NTI and null expectation of zero were tested using two-tailed *T* test at 95% confidence level: if the NTI is significantly higher than zero, this indicates that the community phylogenetic structure should be clustered; if the NTI is lower than zero, the community should be phylogenetically overdispersed; and if the NTI does not differ from zero, the community should be phylogenetically random.

## Results

### Plant and soil properties in different patches

The individual densities (one-way ANOVA, *F* = 174.7, *P*<0.001) and heights (*F* = 5.2, *P* = 0.02) of *L. virgaurea* were gradually increased from LD to HD patches (Table S1 in File S1), suggesting that our selected patches could well represent the spreading degree of *L. virgaurea*. With the spreading of *L. virgaurea*, both species richness (*F* = 6.3, *P* = 0.005) and shoot biomass (*F* = 5.1, *P* = 0.012) of their neighborhood plants were declined significantly (Table S1 in File S1), and these changes were especially remarkable for the forb and grass (Table S2 in File S1). Plant tissue P concentration in both *L. virgaurea* and their neighbors varied among patches, with the highest P concentration synchronously occurred in HD patch (Table S1 in File S1). The lowest soil available N and N/P ratio were both present in HD patch, whereas other soil characters (soil moisture, pH, soil C/N ratio, etc.) were similar across the density gradient of *L. virgaurea* (Table S3 in File S1).

### AM fungal colonization and community composition

**Figure 1:**
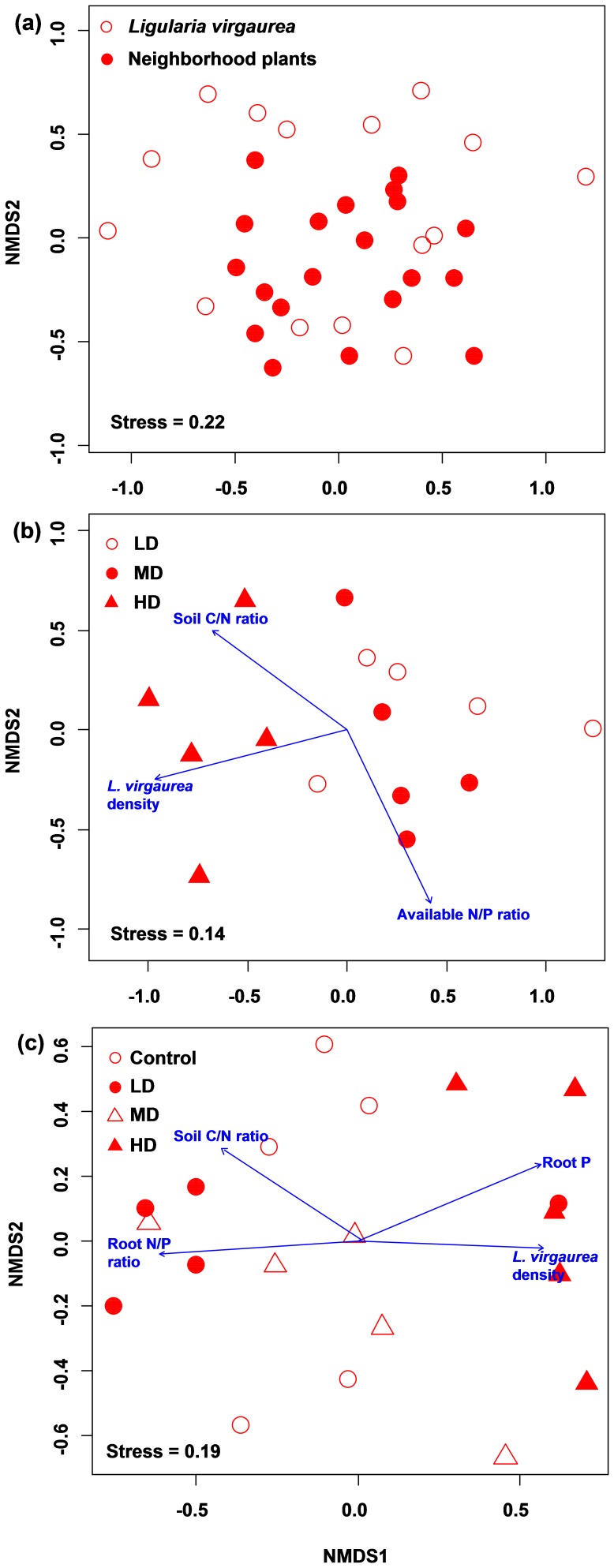
Nonmetric multidimensional scaling (NMDS) ordination of AM fungal communities between *Ligularia virgaurea* roots and neighborhood plant roots (a), in *L. virgaurea* roots (b) and in neighborhood plant roots (c) based on Bray-Curtis dissimilarity. Plant properties and soil characteristics were fitted as vectors onto each ordination plot, and significant vectors at 95% confidence level (*P*≤0.05) were displayed onto the NMDS ordination plots. Control, LD, MD and HD represent no *L. virgaurea*, low density, moderate density and high density of *L. virgaurea*, respectively.

The spreading of *L. virgaurea* significantly changed %AC (*F* = 3.9, *P* = 0.03) and %VC (*F* = 6.3, *P* = 0.01) in their neighborhood plant roots, but did not affect the AM colonization in the roots of themselves (Table S4 in File S1). A total of 324 AM fungal sequences (74.5% of total sequences), based on 1184 AM fungal clones, were obtained in this study. These sequences could be delimited into 30 phylotypes with sequence identity ≥97% (Table 1, Figure S1 in File S1). Of these phylotypes, seven were related to sequences of described species, six to spore-derived sequences, fifteen to uncultured AM fungi, and the remaining two phylotypes (Glo-14 and Aca-17) were undescribed previously (<97% identity with published sequences; Figure S1 in File S1). These phylotypes belonged to 10 Glomeromycotan families: fourteen Glomeraceae, four Acaulosporaceae, four Claroideoglomeraceae, two Archaeosporaceae, one Gigasporaceae, one Ambisporaceae, one Pacisporaceae, one Diversisporaceae, one Geosiphonaceae and one Paraglomeraceae phylotype (Figure S1 in File S1). Sampling effort curves clearly showed that the majority of AM fungal phylotypes in both root systems were sufficiently identified (Figure S2 in File S1). In total, 26 and 23 AM fungal phylotypes were detected in *L. virgaurea* and neighborhood plant roots, respectively, and 18 phylotypes were same in both root systems (Table 1). In addition, the community compositions of AM fungi in both root systems were similar (Figure 1a) and correlated with each other significantly (Mantel *r* = 0.57, *P* = 0.001).

**Table 1:** Relative abundance (%, proportion of clone numbers) of each arbuscular mycorrhizal fungal phylotype and indicator species in the roots of *Ligularia virgaurea* and its neighborhood plants in different patches.

	Related species or sequences	*Ligularia virgaurea*			Neighborhood plants			
		LD	MD	HD	Control	LD	MD	HD
Glo-1(KF467261^a^)	u. *Glomus*	0	0	4.9	0	0	0	5.4
Glo-2 (KF467262)	*Glomus* sp.	6.7	10.1	7.0	13.6	16.7	26.8	37.0
Glo-3 (KF467303)	u. *Glomus*	5.2	0	9.8	0	3.0	0.6	0
Glo-4 (KF467276)	u. *Glomus*	9.0	2.5	0	1.1	14.3	6.7	0
Glo-5 (KF467281)	*Glomus* sp.	0	0	1.4	0	0	0	0
Glo-6 (KF467270)	u. *Glomus*	3.7	1.9	0	3.4	3.4	1.7	0
Glo-7 (KF467255)	*Glomus* sp.	0	0	0	0	2.0	0	6.0
Glo-8 (KF467256)	u. *Glomus*	9.0	1.9	5.6	14.1	1.0	5.0	10.9
Glo-9 (KF467269)	u. *Glomus*	7.5	**52.8** c	0.7	11.9	12.8	24.0	0
Glo-10 (KF467259)	*Glomus indicum*	0	1.3	0	0	1.5	0.6	**8.7** c
Glo-11 (KF467271)	u. *Glomus*	21.6	0	0	0	10.3	3.4	0
Glo-12 (KF467288)	u. *Glomus*	9.7	1.9	0	13.6	20.2	10.1	4.9
Glo-13 (KF467266)	u. *Glomus*	1.5	0	5.6	0.6	0	0	2.2
Glo-14 (KF467282)	u. *Glomus* (new^b^)	0	0	30.1	2.3	1.5	2.2	1.6
Aca-15 (KF467264)	*Acaulospora* sp.	3.0	0	0	3.4	0	0	1.1
Aca-16 (KF467257)	*Acaulospora* sp.	0	0	0	0	0	0	0.5
Aca-17 (KF467260)	u. *Acaulospora* (new)	0	0	0	0	0	0	0.5
Aca-18 (KF467263)	*Acaulospora laevis*	0	2.5	0	0	0	0	3.8
Ent-19 (KF467274)	*Entrophospora baltica*	0	0	0.7	0	0	0	0
Pac-20 (KF467280)	*Pacispora scitillans*	0	0	1.4	0	0	0	0
Scu-21 (KF467268)	*Scutellospora calospora*	0	0	3.5	0	0	0	0
Div-22 (KF467267)	*Diversisipora* sp.	3.0	0.6	9.1	7.3	0	0.6	6.0
Cla-23 (KF467265)	*Claroideoglomus lamellosum*	9.0	5.0	4.2	5.1	0	3.9	3.3
Cla-24 (KF467277)	u. *Claroideoglomus*	2.2	0	0	0	0	0	0
Cla-25 (KF467258)	u. *Claroideoglomus*	8.2	9.4	0	7.3	13.3	12.3	6.5
Cla-26 (KF467272)	u. *Claroideoglomus*	0.7	8.8	0	0	0	0	0
Arc-27 (KF467279)	*Archaeospora schenckii*	0	0	11.2	0	0	1.7	0
Amb-28 (KF467320)	u. *Ambispora*	0	0	4.9	16.4	0	0.6	1.1
Geo-29 (KF467324)	u. *Geosiphon*	0	0	0	0	0	0	0.5
Par-30 (KF467275)	u. *Paraglomus*	0	1.3	0	0	0	0	0

Control, LD, MD and HD represent no *L. virgaurea*, low density, moderate density and high density of *L. virgaurea*,, respectively; u: uncultured.

a The GenBank accession number of the representative sequence.

b ‘new’ in parentheses indicates <97% similarity with published sequences in the GenBank database.

c Bold font indicates that the phylotype is a significant (Indval≥0.6, *P*≤0.05) indicator species for a particular patch.

Spreading of *L. virgaurea* did not change the AM fungal richness and Shannon-Weiner diversity in the roots of *L. virgaurea* (richness: *F* = 0.5, *P* = 0.62; diversity: *F* = 0.9, *P* = 0.43) and neighborhood plants (richness: *F* = 0.2, *P* = 0.79; diversity: *F* = 0.3, *P* = 0.80). However, the community compositions of AM fungi in both root systems varied among patches (Figure 1b,c). An average of 42% (neighborhood plant roots) and 48% (*L. virgaurea* roots) abundance per sample belonged to one phylotype, and the most dominant phylotypes in each root system-patch combination were different, with exception of the phylotype Glo-2, which was predominant in neighborhood plant roots in both MD and HD patches (Table 1). In addition, some AM fungal phylotypes were only detected in a particular patch or root system-patch combination; for example, the Glo-1 was specific for HD patch, and Scu-21 was only detected in *L. virgaurea* roots in HD patch (Table 1). Indicator species were also different between AM fungal communities in two root systems; for example, Glo-10 was the indicator species for neighborhood plants in HD, but Glo-9 for *L. virgaurea* in MD (Table 1).

The community composition of AM fungi in *L. virgaurea* roots was significantly related with *L. virgaurea* density (*r^2^* = 0.6, *P* = 0.004), soil available N/P ratio (*r^2^* = 0.57, *P* = 0.01) and soil C/N ratio (*r^2^* = 0.43, *P* = 0.03); of which, the *L. virgaurea* density was the most relevant variable (Figure 1b). Some soil and plant variables were also correlated with the AM fungal communities colonizing neighborhood plant roots (Figure 1c), and the root N/P ratio (*r^2^* = 0.45, *P* = 0.003) was the most relevant variable, followed by root P (*r^2^* = 0.39, *P* = 0.01) and *L. virgaurea* density (*r^2^* = 0.37, *P* = 0.02). Mantel test revealed that the plant species composition was significantly correlated with AM fungal communities in both root systems (Table 2). P availability was only related with AM fungal community in neighborhood plant roots, while N availability and soil characteristics were not correlated with both fungal communities (Table 2).

**Table 2:** Mantel tests between matrices of AM fungal community in the roots of *Ligularia virgaurea* and its neighborhood plants and matrices of N and P variables, plant community and soil characteristics.

Matrices	Component variables	*Ligularia virgaurea* roots		Neighborhood plant roots	
		Mantel *r*	*P* value	Mantel *r*	*P* value
Plant community	Species compositions	**0.21**	**0.049**	**0.24**	**0.009**
P availability	Soil AP, shoot P, root P	0.18	0.11	**0.23**	**0.019**
N availability	Soil AN, shoot N, root N	0.08	0.263	0.03	0.371
Soil characteristics	All measured soil variables	0.18	0.084	−0.04	0.682

### Neutral model fitting, variation partitioning and phylogenetic analysis of AM fungal community

**Figure 2:**
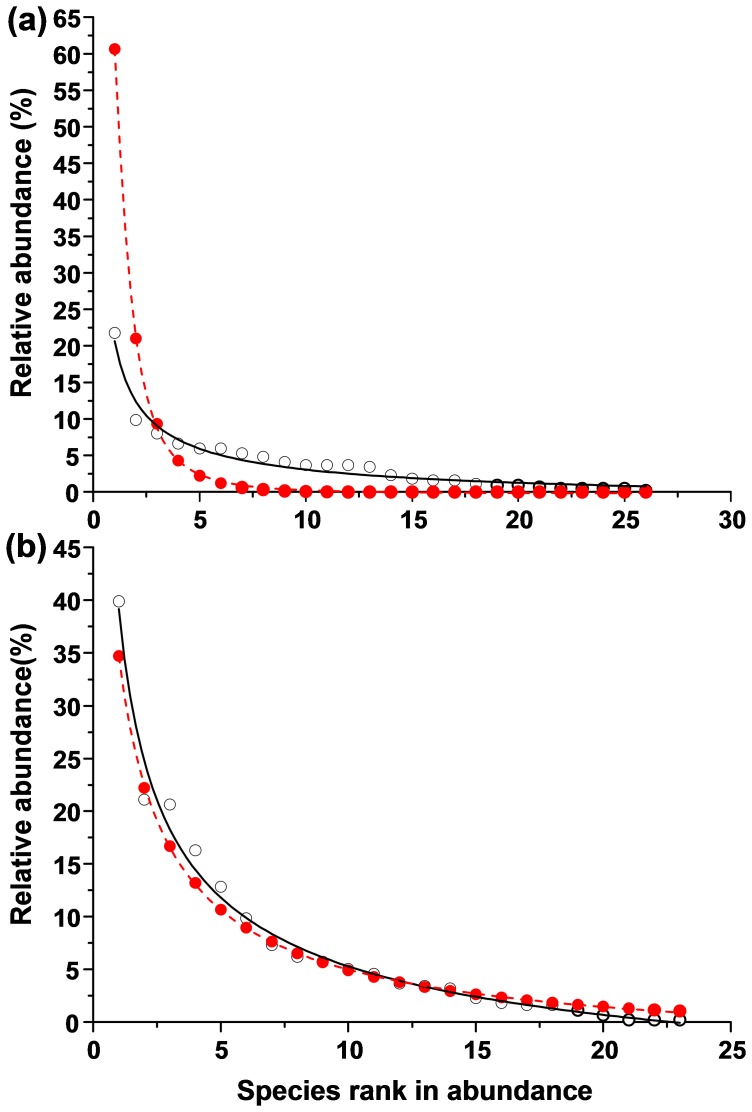
The nonlinear curve fitting of AM fungal abundance versus species rank in abundance in the roots of *Ligularia virgaurea* (a, *χ^2^* = 9.4, *df* = 6, *P* = 0.15; Kolmogorov-Smirnov *Z* = 0.47, *P* = 0.98; estimated parameters, θ = 1.2, I = 7.5) and neighborhood plants (b, *χ^2^* = 8.3, *df* = 4, *P* = 0.33; Kolmogorov-Smirnov *Z* = 0.40, *P* = 0.99; estimated parameters, θ = 9.3, I = 3.8). Open circles and solid line represent observed values and their fitted curve, respectively; filled circles and dotted line represent expected values under a neutral model and their fitted curve, respectively.

We plotted the association between AM fungal relative abundance and species rank in abundance as predicted by a neutral model to test stochastic process in driving community assembly (Figure 2). The abundance distribution of our AM fungal phylotypes in the roots of *L. virgaurea* (*χ^2^* = 9.4, *df* = 6, *P* = 0.15; Kolmogorov-Smirnov *Z* = 0.47, *P* = 0.98; estimated parameters, θ = 1.2, I = 7.5) and neighborhood plants (*χ^2^* = 8.3, *df* = 4, *P* = 0.33; Kolmogorov-Smirnov *Z* = 0.40, *P* = 0.99; estimated parameters, θ = 9.3, I = 3.8) showed a close fitting to the neutral model.

Analysis of variation partitioning explained 21.8% of the variation of AM fungal community composition, based on adjusted *R^2^* value (adj. *R^2^*), in the roots of *L. virgaurea*. Of these variation, 17.5% (adj. *R^2^*_a+b_ = 0.175) was attributed to environmental variables, and 4.4% (adj. *R^2^*_b+c_ = 0.044) to spatially structured variation; moreover, environmental and spatial variables jointly explained 17.3% of the variation of AM fungal community composition in the roots of neighborhood plants; of which, 16.9% (adj. *R^2^*_a+b_ = 0.169) and 9.1% (adj. *R^2^*_b+c_ = 0.091) were attributed to environmental and spatial factors, respectively.

**Figure 3:**
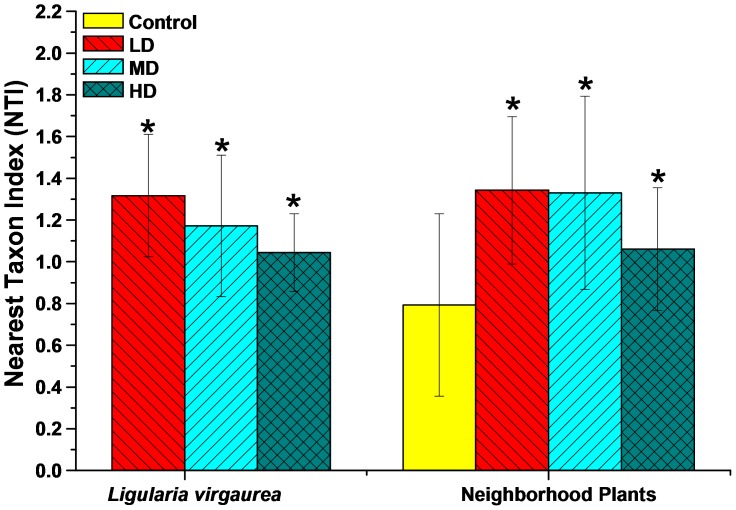
Nearest taxon index (NTI, mean ± SE) of arbuscular mycorrhizal fungal community in the roots of *Ligularia virgaurea* and its neighborhood plants in different patches. Mean NTI is significantly higher than zero, indicating that the community is more phylogenetically clustered than expected by chance. Asterisk indicates that the NTI has significant differences with zero at 95% confidence level (*P*≤0.05). Control, LD, MD and HD represent no *L. virgaurea*, low density, moderate density and high density of *L. virgaurea*, respectively.

Our phylogenetic analysis successfully revealed the phylogenetic patterns of AM fungal community in each patch. The NTI in three patches (LD, MD and HD) was significantly higher than zero in both neighborhood plant roots and *L. virgaurea* roots, whereas it was not significantly different from zero in Control patch (Figure 3). These results suggest that AM fungal communities in LD, MD and HD patches were phylogenetically clustered, and the community in Control patch was phylogenetically random. Although not statistically significant, the NTI for both root systems declined gradually from LD to HD (Figure 3).

## Discussion

In the present study, we did not observe significant changes of AM fungal richness and diversity during the spreading of *L. virgaurea*. However, a total of seven AM fungal phylotypes were only present in HD patch, suggesting that these fungi might be immigrants. It is possible that these immigrated fungi might move from deep soil layer [62] or be introduced from the excrements of soil arthropods [63]. This speculation is also supported by our analysis of AM fungal communities: the community composition in HD was especially distinct with that in Control, LD and MD patches, whilst there was less difference among the later three patches. These findings partly support our hypothesis, and are in line with previous studies, in which some forbs could change the community composition of AM fungi colonizing their surrounding plants [21], [64]. In addition, significant changes of most soil and plant variables only occurred in the HD but not in other patches. This might be another explanation for the distant community composition between HD with others, and indicates that the AM fungal community would be largely changed when the individual density of *L. virgaurea* exceeds a threshold value.

Our Mantel test revealed that the community composition of AM fungi was highly correlated with aboveground plant community, highlighting the importance of plant species composition in regulating their associated fungal community. Furthermore, some soil characteristics (e.g. soil C/N ratio and P availability) were also related with AM fungal communities in both *L. virgaurea* and neighborhood plant roots, suggesting that abiotic factors could also exert effects on the mycorrhizal symbionts. These findings agree with many previous studies showing that the AM fungal communities were determined by both plant communities [65] and some environmental factors [13], [14], and highlight the importance of deterministic process based on niche differentiation in driving the community assembly of AM fungi. In fact, changes of plant community and soil properties in this study should be ultimately attributed to the spreading of *L. virgaurea*, because its spreading could suppress the seedling recruitment of neighborhood plants by allelochemicals [36] as well as change soil characteristics via changing the activities of soil enzymes [66]. Further studies are encouraged to address how *L. virgaurea* spreading directly and indirectly regulates the AM fungal assemblages in alpine meadow ecosystems.

Our AM fungal communities were typically over-dominated by a single phylotype, and these dominant phylotypes varied in most patch-root system combinations. Similar results were also found in other studies, showing the importance of priority effects in fungal “meta-community” assembly [23], [24]. It has been suggested that the overdominance of AM fungi in a community might result from a positive feedback occurring during the build-up of the plant-AM fungal community [23]. That is, a ‘founder AM fungal species’ colonized plant roots during earlier community succession would benefit from more plant derived-carbon than “latecomers”, which would favor its growth and spreading through the soil, and increase its probability of colonizing newly formed roots. Our neutral model fitting also confirms the role of stochastic process in structuring AM fungal community. Collectively, our results corroborate other findings [16], [67], [68], and suggest that deterministic and stochastic processes jointly determine the community assembly of AM fungi. In our case, however, a stronger effect of environmental component on the community composition was demonstrated by the variation partitioning, indicating that the effect of deterministic process was stronger than stochastic process in structuring AM fungal community during the spreading of *L. virgaurea*.

It is interesting that the AM fungal communities in patches with absence (Control) and presence (LD to HD) of *L. virgaurea* were phylogenetically random and clustered, respectively. These results suggest that the community assembly of AM fungi in patch without *L. virgaurea* was mainly determined by stochastic process, but once this toxic plant was present, the environmental filtering became as a major process in structuring fungal communities. Competitive exclusion can also produce phylogenetically clustered pattern even when the functional traits is evolutionally conserved [69], but in our case, the shift of phylogenetic pattern should be attributed to environmental filtering from the allelochemicals secreted by *L. virgaurea*, because the major difference between Control and other patches was the presence or absence of *L. virgaurea*. Although we did not measure the effects of *L. virguarea*'s allelochemicals on fungal community, previous studies have shown that the allelochemicals could significantly influence fungal community composition by suppressing the spore germination of some AM fungal species [70] and the formation of mycorrhizal association [39], [40]. It is therefore that the allelochemicals of *L. virgaurea* in soil might be an environmental stress for mycorrhizal fungi, and this can in part explain the phylogenetically random pattern observed in our Control patch, where the AM fungi was free from allelochemicals of *L. virgaurea*. Nonetheless, further researches are required to address how the allelochemicals of *L. virgaurea* affect the assemblage and functioning of AM fungal community, and only by which can we fully understand whether the spreading of *L. virgaurea* is involving the indirect effects of allelochemicals on mycorrhizal communities of their surrounding plants.

In conclusion, our study shows that the spreading of *L. virgaurea* in an alpine meadow ecosystem could significantly change the assemblage of AM fungi inside their surrounding plant roots. The phylogenetic structure of AM fungal community dramatically changed from phylogenetical random to clustering during the spreading of *L. virgaurea*. The community assembly of AM fungi in patch without *L. virgaurea* was mainly determined by stochastic process, whereas the environmental filtering became as a major process once the *L. virgaurea* was present. Our findings indicate that the spreading of *L. virgaurea* in alpine meadow ecosystems might involve with the mycorrhizal symbionts, and also highlight the importance of phylogenetic relatedness between species in driving AM fungal community composition. Further studies are encouraged to address the functional changes of AM fungal communities during *L. virgaurea* spreading, as well as the feedbacks of changed AM fungal communities on aboveground plant communities.

## Supporting Information

File S1 Contains Table S1, Changes in plant properties for *Ligularia virgaurea* and its neighborhood plant community in different patches. Data are means ± SE (n = 5). Significant differences among patches within each variable were tested using Tukey's honestly significant difference test (*P*≤0.05) and are indicated by dissimilar letters. Table S2, Changes in plant richness and shoot biomass in different patches. Data are means ± SE (n = 5). Significant differences among patches within each variable were tested using Tukey's honestly significant difference test (*P*≤0.05) and are indicated by dissimilar letters. Table S3, Soil characteristics in different patches. Data are means ± SE (n = 5). Significant differences among patches within each variable were tested using Tukey's honestly significant difference test (*P*≤0.05) and are indicated by dissimilar letters. Table S4, Root length colonization (RLC%), arbuscular colonization (AC%), and vesicular colonization (VC%) colonized by AM fungi in the roots of *Ligulaira virgaurea* and its neighborhood plants in different patches. Data are means ± SE (n = 5). Significant differences among patches within each variable were tested using Tukey's honestly significant difference test (*P*≤0.05) and are indicated by dissimilar letters. Figure S1, Neighbor-joining phylogenetic tree inferred from representative AM fungal 18S rRNA gene sequences (with bold font) of each phylotype (Glo-1 etc.) identified in this study and reference sequences from GenBank. Numbers above the branches are credibility values (values ≥70% are shown). Figure S2, Sampling effort curves (Mao Tau) for AM fungi detected in both *Ligularia virgaurea* roots (a) and neighborhood plant roots (b) in all patches. Control, LD, MD and HD represent no *L. virgaurea*, low density, moderate density and high density of *L. virgaurea*, respectively.
(DOC)
