# Supplementary material for: Relative Importance of Deterministic and Stochastic Processes in Driving Arbuscular Mycorrhizal Fungal Assemblage during the Spreading of a Toxic Plant
Source: PLoS One. 2014 Apr 18;9(4):e95672. doi: 10.1371/journal.pone.0095672 (PMC3991703; doi:10.1371/journal.pone.0095672)
Supplement: File S1 — Contains Table S1, Changes in plant properties for Ligularia virgaurea and its neighborhood plant community in different patches. Data are means ± SE (n = 5). Significant differences among patches within each variable were tested using Tukey's honestly significant difference test (P≤0.05) and are indicated by dissimilar letters. Table S2, Changes in plant richness and shoot biomass in different patches. Data are means ± SE (n = 5). Significant differences among patches within each variable were tested using Tukey's honestly significant difference test (P≤0.05) and are indicated by dissimilar letters. Table S3, Soil characteristics in different patches. Data are means ± SE (n = 5). Significant differences among patches within each variable were tested using Tukey's honestly significant difference test (P≤0.05) and are indicated by dissimilar letters. Table S4, Root length colonization (RLC%), arbuscular colonization (AC%), and vesicular colonization (VC%) colonized by AM fungi in the roots of Ligulaira virgaurea and its neighborhood plants in different patches. Data are means ± SE (n = 5). Significant differences among patches within each variable were tested using Tukey's honestly significant difference test (P≤0.05) and are indicated by dissimilar letters. Figure S1, Neighbor-joining phylogenetic tree inferred from representative AM fungal 18S rRNA gene sequences (with bold font) of each phylotype (Glo-1 etc.) identified in this study and reference sequences from GenBank. Numbers above the branches are credibility values (values ≥70% are shown). Figure S2, Sampling effort curves (Mao Tau) for AM fungi detected in both Ligularia virgaurea roots (a) and neighborhood plant roots (b) in all patches. Control, LD, MD and HD represent no L. virgaurea, low density, moderate density and high density of L. virgaurea, respectively. (DOC) [file pone.0095672.s001.doc]

**Supporting Information**

**Table S1** Changes in plant properties for *Ligularia virgaurea* and its neighborhood plant community in different patches. Data are means ± SE (n = 5). Significant differences among patches within each variable were tested using Tukey’s honestly significant difference test (*P* ≤ 0.05) and are indicated by dissimilar letters.

|  | *Ligularia virgaurea* | | | | |  | Neighborhood plant community | | | |
| --- | --- | --- | --- | --- | --- | --- | --- | --- | --- | --- |
|  | Individual  density (0.25 m^-2^) | Individual  height (cm) | Shoot biomass  (g 0.25 m^-2^) | Shoot P  (mg.g^-1^) | Root P  (mg.g^-1^) |  | Species  richness | Shoot biomass  (g 0.25 m^-2^) | Shoot P  (mg.g^-1^) | Root P  (mg.g^-1^) |
| Control | _ | _ | _ | _ | _ |  | 21.0 ± 1.7a | 86.8 ± 12.0a | 1.9 ± 0.1ab | 1.2 ± 0.1ab |
| LD | 8.8 ± 2.7c | 30.8 ± 2.8b | 24.9 ± 4.4c | 1.7 ± 0.1ab | 1.2 ± 0.1b |  | 21.0 ± 1.1a | 47.4 ± 4.1b | 1.5 ± 0.1b | 1.0 ± 0.1b |
| MD | 26.2 ± 1.4b | 39.8 ± 3.0ab | 69.7 ± 4.0b | 1.6 ± 0.1b | 1.2 ± 0.1b |  | 20.4 ± 1.2a | 51.3 ± 7.9ab | 1.6 ± 0.1b | 1.1 ± 0.1b |
| HD | 49.0 ± 2.2a | 43.6 ± 1.8a | 92.4 ± 13.6a | 2.0 ± 0.1a | 1.6 ± 0.1a |  | 15.6 ± 0.7b | 54.3 ± 10.5ab | 2.0 ± 0.2a | 1.5 ± 0.2a |

Control, LD, MD and HD represents no *L. virgaurea*, low density, moderate density and high density of *L. virgaurea*, respectively.

**Table S2** Changes in plant richness and shoot biomass in different patches. Data are means ± SE (n = 5). Significant differences among patches within each variable were tested using Tukey’s honestly significant difference test (*P* ≤ 0.05) and are indicated by dissimilar letters.

|  | Plant species richness | | | | |  | Shoot biomass (g 0.25 m^-2^) | | | | |
| --- | --- | --- | --- | --- | --- | --- | --- | --- | --- | --- | --- |
|  | Sedge | Legume | Grass | forb | Total |  | Sedge | Legume | Grass | forb | Total |
| Control | 2.0 ± 0 | 0.6 ± 0.4 | 3.2 ± 0.5 | 16.2 ± 0.9ab | 22.0 ± 1.1a |  | 26.4 ± 10.3 | 0.1 ± 0.1 | 25.0 ± 5.9a | 35.2 ± 3.5b | 86.7 ± 11.9ab |
| LD | 2.0 ± 0 | 0.2 ± 0.2 | 2.4 ± 0.7 | 17.4 ± 0.9a | 22.0 ± 1.2a |  | 8.1 ± 4.0 | 0.1 ± 0.1 | 13.3 ± 5.4a | 50.7 ± 6.7b | 72.2 ± 7.3b |
| MD | 2.0 ± 0 | 1.0 ± 0.5 | 2.8 ± 0.4 | 15.8 ± 1.2ab | 21.6 ± 1.3a |  | 11.3 ± 3.0 | 0.2 ± 0.1 | 17.5 ± 5.5a | 91.9 ± 5.9 a | 120.9 ± 11.3ab |
| HD | 2.0 ± 0 | 0.2 ± 0.2 | 1.2 ± 0.4 | 13.0 ± 0.3b | 16.4 ± 0.9b |  | 26.3 ± 9.4 | 0.1 ± 0.1 | 5.3 ± 2.6b | 115.0 ± 15.6 a | 146.7 ± 23.7a |

Control, LD, MD and HD represents no *L. virgaurea*, low density, moderate density and high density of *L. virgaurea*, respectively.

**Table S3** Soil characteristics in different patches. Data are means ± SE (n = 5). Significant differences among patches within each variable were tested using Tukey’s honestly significant difference test (*P* ≤ 0.05) and are indicated by dissimilar letters.

|  | Soil moisture  (%) | pH | Available N  (mg.kg^-1^) | Available P  (mg.kg^-1^) | Available  N/P ratio | Total N  (%) | Organic C  (%) | Soil C/N  ratio |
| --- | --- | --- | --- | --- | --- | --- | --- | --- |
| Control | 35.5 ± 2.10 | 6.3 ± 0.24 | 25.6 ± 3.05 ab | 4.3 ± 0.98 | 6.9 ± 1.52ab | 0.5 ± 0.06 | 3.3 ± 0.37 | 6.7 ± 0.07 |
| LD | 39.2 ± 1.90 | 6.3 ± 0.22 | 23.8 ± 1.22 ab | 2.8 ± 0.14 | 8.7 ± 0.71ab | 0.5 ± 0.02 | 3.2 ± 0.12 | 6.7 ± 0.04 |
| MD | 36.1 ± 1.23 | 6.1 ± 0.20 | 34.7 ± 4.39 a | 3.2 ± 0.38 | 11.2 ± 1.04 a | 0.6 ± 0.07 | 3.7 ± 0.43 | 6.7 ± 0.05 |
| HD | 41.3 ± 2.54 | 6.0 ± 0.20 | 22.2 ± 1.55 b | 3.9 ± 0.57 | 6.2 ± 1.11 b | 0.6 ± 0.07 | 4.2 ± 0.48 | 6.9 ± 0.04 |

Control, LD, MD and HD represents no *L. virgaurea*, low density, moderate density and high density of *L. virgaurea*, respectively.

**Table S4** Root length colonization (RLC%), arbuscular colonization (AC%), and vesicular colonization (VC%) colonized by AM fungi in the roots of *Ligulaira virgaurea* and its neighborhood plants in different patches*.* Data are means ± SE (n = 5). Significant differences among patches within each variable were tested using Tukey’s honestly significant difference test (*P* ≤ 0.05) and are indicated by dissimilar letters.

|  | *Ligularia virgaurea* roots | | |  | Neighborhood plant roots | | |
| --- | --- | --- | --- | --- | --- | --- | --- |
|  | AC (%) | VC (%) | RLC (%) |  | AC (%) | VC (%) | RLC (%) |
| Control | _ | _ | _ |  | 14.7 ± 4.72a | 5.2 ± 1.48b | 45.5 ± 3.36 |
| LD | 1.4 ± 1.27 | 5.5 ± 2.76 | 54.7 ± 11.75 |  | 3.9 ± 1.40b | 14.5 ± 1.77ab | 48.0 ± 3.14 |
| MD | 0.1 ± 0.11 | 4.1 ± 0.89 | 50.3 ± 8.39 |  | 7.8 ± 1.47ab | 22.3 ± 5.02a | 65.3 ± 5.48 |
| HD | 1.6 ± 0.68 | 1.9 ± 0.90 | 34.1 ± 8.32 |  | 3.8 ± 0.75b | 10.6 ± 1.56b | 49.7 ± 7.65 |

Control, LD, MD and HD represents no *L. virgaurea*, low density, moderate density and high density of *L. virgaurea*, respectively.

NMDS axis 2

**Figure S1** Neighbor-joining phylogenetic tree inferred from representative AM fungal 18S rRNA gene sequences (with bold font) of each phylotype (Glo-1 etc.) identified in this study and reference sequences from GenBank. Numbers above the branches are credibility values (values ≥ 70% are shown).

**Figure S2** Sampling effort curves (Mao Tau) for AM fungi detected in both *Ligularia virgaurea* roots (a) and neighborhood plant roots (b) in all patches. Control, LD, MD and HD represents no *L. virgaurea*, low density, moderate density and high density of *L. virgaurea*, respectively.
